# System effectiveness of a targeted free mass distribution of long lasting insecticidal nets in Zanzibar, Tanzania

**DOI:** 10.1186/1475-2875-9-173

**Published:** 2010-06-18

**Authors:** Netta Beer, Abdullah S Ali, Don de Savigny, Abdul-wahiyd H Al-mafazy, Mahdi Ramsan, Ali K Abass, Rahila S Omari, Anders Björkman, Karin Källander

**Affiliations:** 1 Division of Global Health (IHCAR), Department of Public Health Sciences, Karolinska Institutet, 171 77 Stockholm, Sweden; 2 Malaria Research Unit, Department of Medicine, Karolinska Institutet, Stockholm, Sweden; 3 Zanzibar Malaria Control Programme (ZMCP), Ministry of Health, Zanzibar, Tanzania; 4 Department of Epidemiology and Public Health, Swiss Tropical and Public Health Institute, Basel, Switzerland and University of Basel, Basel, Switzerland; 5 Research Triangle Institute (RTI), Dar-es-Salaam, Tanzania; 6 Makerere University School of Public Health, Kampala, Uganda; 7 Malaria Consortium Africa, Kampala, Uganda

## Abstract

**Background:**

Insecticide-treated nets (ITN) and long-lasting insecticidal treated nets (LLIN) are important means of malaria prevention. Although there is consensus regarding their importance, there is uncertainty as to which delivery strategies are optimal for dispensing these life saving interventions. A targeted mass distribution of free LLINs to children under five and pregnant women was implemented in Zanzibar between August 2005 and January 2006. The outcomes of this distribution among children under five were evaluated, four to nine months after implementation.

**Methods:**

Two cross-sectional surveys were conducted in May 2006 in two districts of Zanzibar: Micheweni (MI) on Pemba Island and North A (NA) on Unguja Island. Household interviews were conducted with 509 caretakers of under-five children, who were surveyed for socio-economic status, the net distribution process, perceptions and use of bed nets. Each step in the distribution process was assessed in all children one to five years of age for unconditional and conditional proportion of success. System effectiveness (the accumulated proportion of success) and equity effectiveness were calculated, and predictors for LLIN use were identified.

**Results:**

The overall proportion of children under five sleeping under any type of treated net was 83.7% (318/380) in MI and 91.8% (357/389) in NA. The LLIN usage was 56.8% (216/380) in MI and 86.9% (338/389) in NA. Overall system effectiveness was 49% in MI and 87% in NA, and equity was found in the distribution scale-up in NA. In both districts, the predicting factor of a child sleeping under an LLIN was caretakers thinking that LLINs are better than conventional nets (OR = 2.8, p = 0.005 in MI and 2.5, p = 0.041 in NA), in addition to receiving an LLIN (OR = 4.9, p < 0.001 in MI and in OR = 30.1, p = 0.001 in NA).

**Conclusions:**

Targeted free mass distribution of LLINs can result in high and equitable bed net coverage among children under five. However, in order to sustain high effective coverage, there is need for complimentary distribution strategies between mass distribution campaigns. Considering the community's preferences prior to a mass distribution and addressing the communities concerns through information, education and communication, may improve the LLIN usage.

## Background

Malaria kills about one million people and causes up to 250 million clinical episodes every year [1]. Most of the deaths occur in children under five living in sub-Saharan Africa (SSA), where at least 20% of child deaths are due to malaria [2]. The two major measures for malaria control have been prompt and effective treatment and prevention. Bed nets have been used for protection against insect bites long before the discovery that mosquitoes transmitted malaria [3]. In the last decade, there has been a renewed interest in bed nets, and especially insecticide-treated nets (ITN) [2,4]. The Abuja targets, which were declared by African heads of states in 2000, include a clause that at least 60% of those at risk of malaria, particularly children under five years of age and pregnant women, should benefit from protective measures such as ITNs by the year 2005 [5], which was later revised to reach at least 80% coverage by 2010 [6]. However, reports from 18 African countries show that in 2006-2007 only 23% of children slept under ITNs [1]. One of the major challenges with ITNs has been sustaining the necessary bi-annual re-treatment of the nets. In response to this issue, long-lasting insecticidal nets (LLIN), which are factory pre-treated and do not require re-treatment for 3-5 years, were developed [2].

Community-based randomized control trials from countries with varying transmission intensity have shown that ITNs can achieve an average efficacy of 18% reduction in child mortality even with sub-optimal adherence in use [7]. However, the effectiveness of ITNs in a programme setting is expected to be considerably lower [8], as was also demonstrated by Schellenberg *et al *[9]. Beliefs such as ITNs only having partial effectiveness, fear of insecticide toxicity, seasonal variation in the perceived risk of malaria, difficulties in mounting the nets, sleeping arrangements, temporary migrations, young age, and high temperatures are some of the factors that lower ITN use and compromise their effectiveness [10-12]. Other, sometimes more important, factors are found in the health systems that delivers the ITNs, and there has been a debate as to whether ITNs should be provided free of charge through periodic mass or continuous distributions [13], targeted subsidies [14], voucher systems [15], or through social marketing techniques [9].

Additionally, as in other health-related issues, the access to good medical care or preventive measures tend to vary inversely with the need of the population served (the inverse care law) [16]. Thus, the poor, who are sometimes more exposed to mosquitoes and malaria, are often less likely to access anti-malarial treatment [17] and to own or use bed nets [2,18,19]. For an intervention such as ITNs to achieve high effectiveness, systems' barriers which impede optimal coverage need to be mitigated at several different levels [20]. Information on the success rates in each of these steps can highlight which steps are in greatest need of improvement [8,20] and can be used to measure how well an intervention works in a program setting - hereby referred to as *system effectiveness*. The ratio between the system effectiveness of the least poor and poorest populations is defined as *equity effectiveness*, as suggested by Tugwell and de Savigny [20].

A targeted free mass distribution of LLINs to all pregnant women and children under five took place in Zanzibar in 2005-2006. The aim of this study was to determine LLIN effective coverage (children sleeping under an LLIN), system effectiveness, equity effectiveness and predictive factors for LLIN use in children under five, 4-9 months after the distribution took place.

## Methods

### Study area

The study was conducted during May-June 2006 in the Micheweni (MI) and North A (NA) districts of Zanzibar, an archipelago off the coast of mainland Tanzania. Previously the area had high transmission of *Plasmodium falciparum *malaria, but in recent years malaria prevalence has decreased as a result of implementation and reinforcement of artemisinin-based combination therapy (ACT), ITNs and indoor residual spraying [21]. ITN interventions included sensitization campaigns, small scale social marketing efforts and re-treatment campaigns. A cost recovery scheme was implemented from 2003-2005, whereby nets provided by UNICEF were sold at a reduced price at antenatal clinics (Personal communication with Abdullah S. Ali). In May 2005 the overall ITN use in children under five in Zanzibar was documented at 40%, with MI district having the lowest under-five ITN use of less than 10% [22]. As a result of these low figures, retreatment campaigns were carried out in MI district during 2005.

### The distribution process

The Global Fund to fight AIDS, Tuberculosis and Malaria (GFATM) and the President's Malaria Initiative (PMI) supported the Zanzibar Malaria Control Programme (ZMCP) to carry out a targeted free mass distribution campaign to all pregnant women and children under five, which took place from August 2005 till February 2006. The distributed nets were blue rectangular Olyset^® ^nets, which are made of polyethylene and have a mesh size of 4 × 4 mm. Since MI district had the lowest ITN use, it was chosen as the site for trial implementation in August 2005 (during the dry season). The distribution scale-up in the other districts followed in January 2006 (during the short rains season) after operational reports and focus group discussions (FGDs) conducted in MI had been reviewed and ways of improving the distribution process were addressed. The distribution for children under five was completely different from that for the pregnant women - pregnant women were registered and given an LLIN in a separate process through antenatal clinics. This article focuses only on the children's distribution.

For children under five, the distribution process included registration of all eligible children through house-to-house visits conducted by local leaders (shehas) representatives. Thereafter, the distribution dates and locations were announced through mass media and village criers, and caretakers arrived at the distribution point to collect their children's nets.

In the trial phase, only public health facilities were used as distribution points which made the distribution last several weeks in each shehia (the smallest administrative unit which comprises several villages). The LLINs given during the trial phase did not include any instructions or information for the users and there was an insufficient number of LLINs for all registered children to receive a net. In the distribution scale-up, more distribution points were used which shortened the distribution to only 1-2 days in each shehia. Other improvements were including information and instructions with simple written and pictorial illustrations of how to use the net, increasing the amount of nets available and, in addition to the household registration, using an under-five population projection to estimate the need. The information, education and communication (IEC) strategies had also improved in the distribution scale-up which was part of the "Kataa Malaria" (reject malaria) PMI campaign. In all, 191,537 LLINs were distributed to children under five, and 18,432 LLINs were given to pregnant women. This distribution was the first source of LLINs in Zanzibar.

### Sampling and sample size

This was a cross sectional household survey in which a two-stage cluster sampling technique [23] was used to randomly select 22 sampling units (shehias) from which households were randomly selected using a household list and in proportion to shehia size, as described by Bhattarai *et al *[21]. Assuming a proportion of 50% of children under five sleeping under an LLIN and accounting for a cluster effect of 2, a sample size of 192 households of children under five was needed from each survey in the two districts to determine coverage with an absolute precision of ± 10% and a 95% confidence interval. Since the survey was done in conjunction with the annual cross sectional surveys conducted by Karolinska Institutet and ZMCP in the two districts, the total number of households interviewed was larger than needed (509 households in total). If a household on the sampling list could not be found, or consent could not be obtained, it was replaced by another household from a reserve list. Compliance of community members to participating in the survey was generally good.

### Data collection

In order to assist the operational assessment of the trial distribution in MI district, and to inform the design of the quantitative study questionnaire, five focus group discussions (FGD) were conducted in MI district by a team of four, including a moderator, observer, the lead author and a translator. The FGDs were conducted with mothers (two FGDs), fathers (two FGDs) and health workers (one FGD) and each included 8-12 participants who were purposefully chosen based on their participation in the distribution. The FGDs explored perceptions on LLINs and other nets, as well as opinions, concerns and experiences from the distribution process. The FGDs revealed that many community members did not know that the nets were treated with insecticide. Features of the LLINs were liked by the community were their light blue colour, strength, and ability to keep mosquitoes away, while other features were considered problematic such as the large mesh size and the insufficient height of the nets. Many reported not receiving any information on the LLINs during the distribution, and health workers mentioned the need for sustainable net distribution. The results of the FGDs were quantified in the structured questionnaire. The questionnaire was translated into Kiswahilli, pilot tested and corrected according to the pilot's results.

The quantitative survey was carried out four and nine months after the distribution took place in NA and MI, respectively in the rainy season. During the household interview, a caretaker, preferably the mother was interviewed by one of the 14 trained interviewers, health professionals who had under gone one-week training. The caretakers were asked about bed net ownership and use, experiences with the LLIN distribution, perceptions on LLINs and other bed nets, as well as household characteristics. In addition to asking about bed net usage, the interviewers asked to see the nets reported, and noted whether the nets were hanging above a sleeping space.

The study was approved by the Zanzibar Medical Research Task Force. District leaders and shehas were informed about the study, and before starting the interviews, respondents signed an informed consent form.

### Data analysis

Data was single entered in CSPRO 3.2 by a data entry clerk, checked for consistency and errors, and analyzed in STATA 10. Frequencies and proportions of success in the different steps of the LLIN distribution were computed for eligible children who were above one year old. This is to avoid including children who were not born at the time of registration. Additionally, only children with complete information for all four steps were included.

Four steps of the LLIN distribution process were analyzed: 1) child being registered, 2) caretaker of the child arriving at the distribution point, 3) caretaker of the child receiving an LLIN and 4) child sleeping under an LLIN. Three measures were calculated for each of these four steps, as previously described by Krause et al [24]. The first measure is the *unconditional proportion *(UP) at a defined step, which represents the proportion of under-fives who succeeded in this step, without taking previous steps into account - for example, the percentage of those who received an LLIN out of all eligible children in the survey.

The second measure, called *conditional proportion *(CP), expresses the proportion of under-fives who succeed in a defined step only among those that also succeeded in all the previous steps - for example, the proportion of those who received an LLIN only among those who got registered and had arrived at the distribution point.

The third measure, the *accumulated proportion *(AP) at a certain step, represents the proportion that succeeded in all steps up to, and including, the step concerned out of all under-fives in the survey - for example, the proportion that succeeded to get registered, arrive at distribution point and receive an LLIN out of all under-fives in the study. The AP is a product of all CPs, including the step in question. Each of these three measures (UP, CP and AP) was calculated for every step in the distribution process. The AP of all four steps is defined as the *system effectiveness*.

An asset index was created by principal component analysis (PCA) as suggested by Filmer and Pritchett [25]. Assets which were used in the final model were type of floor, walls and roof, sources of water, and owning a mat, cupboard, sofa, clock, iron, phone, radio, motorcycle, car, TV and fridge. The study population was grouped into socio-economic quintiles based on their asset index. Significance of the differences between the quintiles were calculated using χ^2 ^with *P*-values adjusted for cluster effects at household and shehia level using the STATA svy command. The equity effectiveness was calculated as the ratio of system effectiveness in the least poor and poorest quintiles.

Determinants of LLIN use, a dichotomous variable, were identified using bivariate logistic regression. All variables with a *p*-value ≤ 0.25 in the bivariate analysis were included in the multiple logistic regression model, after they were checked for colinearity. Interaction was tested for between all variables that remained significant in the multivariate model.

## Results

A total of 509 caretakers of children under five were interviewed from 245 households in MI and 264 in NA. The majority of households were headed by men (84%), 44% of the heads of household had no formal education and only 31% had over seven years of education. The majority of the respondents were mothers (79%). The households had between one and three children under five, with a median of one child per household. Overall, information on 787 children under five was obtained, 48% (381/787) male and 52% (406/787) female. The mean age of the children under five was 28 months (SD ± 16).

**Figure 1:**
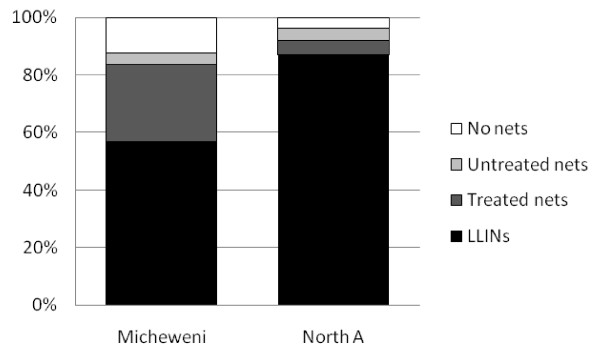
**Proportion of children under five sleeping under different types of nets in Micheweni and North A districts**.

The overall proportion of children under five sleeping under any type of treated net was 83.7% (318/380) in MI and 91.8% (357/389) in NA. The LLIN usage was 56.8% (216/380) in MI and 86.9% (338/389) in NA (Figure 1). Usage among children under five who had received an LLIN in the distribution campaign was also higher in NA (91.6%; 304/332) than MI (72.3%; 162/224).

**Figure 2:**
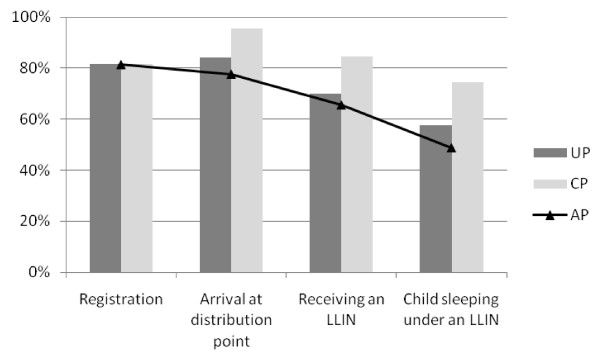
**Unconditional Proportion (UP), Conditional Proportion (CP) and Accumulated Proportion (AP) of the LLIN distribution process in Micheweni**.

**Figure 3:**
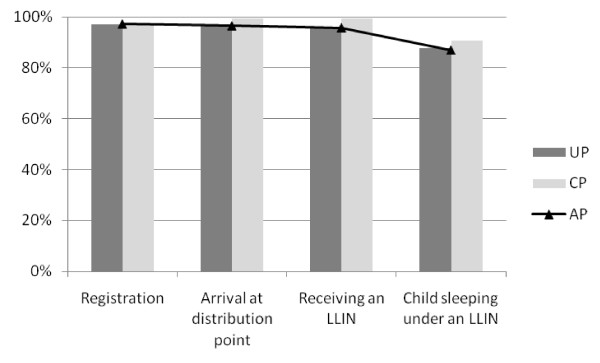
**Unconditional Proportion (UP), Conditional Proportion (CP) and Accumulated Proportion (AP) of the LLIN distribution process in North A**.

The results of the four steps of the distribution process were assessed for 290 children in MI and 284 children in NA, and are shown in Figures 2 and 3. As an example of the interpretation of the study measures, 84.1% (244/290) of the children's caretakers arrived at the distribution point in MI (= UP). Of those who were also registered to receive an LLIN, 95.4% (226/237) arrived at the distribution point (= CP). Hence 77.9% (226/290) were compliant in both steps (= AP).

### Step 1: Being registered to receive an LLIN

Of the eligible children in MI, 81.7% (237/290) were registered to receive an LLIN compared to 97.2% (276/284) of the children in NA. This being the first step in the distribution process, the UP, CP and AP values are the same. The most common explanations given by the caretaker as to why the child had not been registered were that no one had come to register the child (18 cases in MI only), and that the child was not home at the time of registration (15 cases in MI and 3 in NA). Other reasons included the mother not being at home at the time of registration or not having a birth certificate.

### Step 2: Arriving at the distribution point

Overall, 84.1% (244/290) and 96.8% (275/284) of the caretakers arrived to collect LLIN in MI and NA, respectively (UP). Among the children who were registered, 95.4% (226/237) of the caretakers arrived to the distribution point in MI and 99.3% (274/276) in NA (CP). Six of the caretakers of registered children who reported not arriving at the distribution point mentioned reasons such as not being in the area at the time of distribution, and being unable or not having time to come. Among the children who were not registered, 34% (18/53) of the caretakers still arrived at the distribution point in MI, compared to only 12% (1/8) in NA.

### Step 3: Receiving an LLIN

A total of 70% (203/290) of the children in MI and 95.8% (272/284) of the children in NA had received an LLIN in the distribution campaign (UP). Of those who were registered, and whose caretakers arrived at the distribution point, 84.5% (191/226) from MI and 99.3% (272/274) from NA received an LLIN (CP). Of those who were not registered or whose caretakers had not arrived at the distribution point, 19% (12/64) from MI still received an LLIN. In 35 cases in MI and two cases in NA children did not receive an LLIN despite being registered and arriving to collect the net. In MI, the most common reasons for this failure were that only one net was distributed per household (18/35) or because the nets were finished (10/35). In the two cases in NA, it was because the child's name was not found in the lists.

### Step 4: Sleeping under an LLIN

In MI, 57.6% (167/290) had been sleeping under an LLIN compared to 87.7% (249/284) in NA (UP). Of those who were registered, whose caretakers had arrived at the distribution point and who had received an LLIN from the distribution, the proportion who slept under an LLIN was 74.4% (142/191) in MI and 90.8% (247/272) in NA (CP). Among the 87 children in MI and 12 children in NA who did not receive a net from the distribution campaign, 21 (24%) and 2 (17%) still slept under an LLIN, respectively.

All UP and CP steps were higher in NA than in MI. When taking into consideration all the steps in the distribution process, the system effectiveness (AP) was 49% in MI compared to 87% in NA.

### Equity effectiveness

When comparing the distribution steps in the poorest and least poor socio-economic quintiles in MI, significantly fewer of the poorest got registered, arrived at the distribution point and received the LLINs. The largest and most significant difference between the poorest and the least poor was in the registration step (70% vs. 96%, p = 0.0019). The proportion of children sleeping under an LLIN was higher among the poorest in those who completed the previous steps (CP), whereas the overall proportion of children sleeping under the LLIN (UP) was still higher in the least poor. Overall, in MI, system effectiveness was 1.5 times higher in the least poor compared to the poorest (64% vs. 44%) (p = 0.076). In NA the proportions of succeeding in all steps were virtually equal, with no statistically significant differences between the poorest and least poor, resulting in an equity ratio of 1 (Table 1).

**Table 1:** Conditional Proportion (CP), Unconditional Proportion (UP) and Accumulated Proportion (AP) of the distribution steps in the poorest and least poor quintiles.

		Registered	Arrived at distribution point	Received an LLIN	Child sleeping under an LLIN	System effectiveness (AP)	Least poor: poorest equity effectiveness ratio	
**Micheweni**								
Least poor n = 50	CP UP	48/50 (96%) 48/50 (96%)	48/48 (100%) 48/50 (96%)	43/48 (90%) 43/50 (86%)	32/43 (74%) 32/50 (64%)	64%	1.5	(Pro-rich)
Poorest n = 54	CP UP	38/54 (70%) 38/54 (70%)	36/38 (95%) 43/54 (80%)	32/36 (89%) 37/54 (69%)	24/32 (75%) 32/54 (59%)	44%		
								
P-value	CP UP	0.0019 0.0019	0.24 0.019	0.93 0.044	0.95 0.65	0.076		

**North A**								
Least poor n = 67	CP UP	65/67 (97%) 65/67 (97%)	65/65 (100%) 65/67 (97%)	64/65 (98%) 64/67 (96%)	58/64 (91%) 59/67 (88%)	87%	1	(Equitable)
Poorest n = 45	CP UP	43/45 (96%) 43/45 (96%)	43/43 (100%) 43/45 (96%)	43/43 (100%) 43/45 (96%)	39/43 (91%) 39/45 (87%)	87%		
								
P-value	CP UP	0.69 0.69	- 0.69	0.43 0.99	0.99 0.82	0.99		

### Predictors of LLIN use

Receiving an LLIN and thinking LLINs are better than conventional nets were significantly associated with LLIN use in bivariate analysis in both districts. Additionally, liking the mesh size was found significant in MI and was entered in the multivariate analysis, and liking the size of the net was entered into the multivariate analysis in NA. In the multivariate models in both districts, factors associated with LLIN use were receiving an LLIN (OR = 4.9, p < 0.001 in MI and OR = 30.1, p = 0.001 in NA) and thinking that LLINs are better than conventional nets (OR = 2.8, p = 0.005 in MI and OR = 2.5, p = 0.041 in NA) (Tables 2 and 3).

**Table 2:** Potential factors associated with under-fives sleeping under an LLIN in bivariate (crude) and multivariate model (adjusted) ^ψ^, Micheweni district.

Variable	Total n = 290	Use LLINs n = 167 (58)	Crude OR	p-value	Adjusted OR	p-value
Not receiving an LLIN	87	21 (24)	1		1	
Receiving an LLIN	203	146 (72)	8.1	< 0.001**	4.9	< 0.001**
Not liking the LLIN mesh size	78	40 (51)	1		1	
Liking the LLIN mesh size	133	99 (74)	2.8	0.003**	1.8	0.067*
Not thinking that LLINs are better than conventional nets	173	78 (45)	1		1	
Thinking that LLINs are better than conventional nets	117	89 (76)	3.9	< 0.001**	2.8	0.005**

Numbers in brackets are percentages

*P < 0.25; **P < 0.05

^ψ ^Factors which were not found associated with children sleeping under an LLIN (p > 0.25): sex of child, sex of the head of the household, education of head of household, liking the LLIN colour, size, shape and strength and knowing that the LLIN was treated.

**Table 3:** Potential factors associated with under-fives sleeping under an LLIN in bivariate (crude) and multivariate model (adjusted) ^ψ^, North A district.

Variable	Total n = 284	Use LLINs n = 249 (88)	Crude OR	p-value	Adjusted OR	p-value
Not receiving an LLIN	12	2 (17)	1		1	
Receiving an LLIN	272	247 (91)	49.4	< 0.001**	30.1	0.001**
Not liking the LLIN size	47	39(83)	1		1	
Liking the LLIN size	224	205 (92)	2.2	0.078*	1.8	0.266
Not thinking that LLINs are better than conventional nets	76	59 (78)	1		1	
Thinking that LLINs are better than conventional nets	208	190 (91)	3	0.002**	2.5	0.041**

Numbers in brackets are percentages

*P < 0.25; **P < 0.05

^ψ ^Factors which were not found associated with children sleeping under an LLIN (p > 0.25): sex of child, sex of the head of the household, education of head of household, liking the LLIN colour, mesh size, shape and strength and knowing that the LLIN was treated.

## Discussion

This study shows that the overall effective use of ITNs among children under five years, 4-9 months after the free mass distribution, was 83.7% in Micheweni (MI) and 91.8% in North A (NA) district. These findings demonstrate that Zanzibar has reached the Abuja target of 80% coverage by 2010 [6]. The ITN use in children under five years observed greatly exceeds the rates previously found in Tanga region in Tanzania, where usage was at 36% in 2003-2005 when net distribution was ongoing under routine conditions by the private sector and non-governmental organizations (NGOs) [19], and at 54% in 2008 after the voucher scheme was introduced [18]. In another study that evaluated the impact of the voucher scheme in different regions of Tanzania, the ITN usage among children under five still remained at a low 26% in 2007, one to three years after its initiation [15]. In Kenya, a higher coverage of 67% was achieved three to six months after a mass distribution of ITNs [26]. The usage rate observed in our study is also higher than what was observed in two randomized controlled trials (65-77%) where nets were given out for free [27,28].

The high coverage of ITNs observed in our study may have been due to prior exposure of the population to community sensitization and information, education and communication (IEC) strategies advocating for ITN use. Additionally, as social marketing, small-scale distributions and re-treatment campaigns took place in Zanzibar prior to this distribution, most (69%) households already owned at least one bed net, and were familiar with the concept of sleeping under a bed net. Other reasons for high usage rates could be due to the short duration between the distribution and the evaluation [11] and the fact that the surveys were carried out during the rainy season, when bed net use tends to increase [11,12,28,29]. While bed net usage could have been overestimated due to desirability bias of caretakers, the error is likely to be minor since less than 5% (31/612) of the nets were not seen hanging above a sleeping area at the time of interview. Additionally, information on details from the distribution process may be affected by recall bias, especially in MI where the distribution took place 9 months prior to the survey. This bias is not likely to affect recalling receiving or using an LLIN, but may cause inaccuracies in reporting registration and arrival at the distribution point.

The overall system effectiveness was 49% in MI after the trial distribution and 87% in NA after the distribution scale-up. System effectiveness represents the proportion of children who have successfully progressed through all steps of the distribution process, i.e. the accumulated proportion (AP). For further calculation of community effectiveness of ITNs on child morality, Tugwell and de Savigny [20] suggest multiplying the AP with ITN efficacy, while Lengeler and Snow [8] suggest a simplified model of multiplying the coverage (i.e. proportion of children sleeping under an ITN) with ITN efficacy. Given that the efficacy of ITNs is not known in a setting like Zanzibar where malaria prevalence is low, we refrain from calculating community effectiveness on child mortality, but instead report on *system effectiveness *as an indicator describing the proportion of ITN coverage that is lost due to system and programmatic issues.

The system effectiveness was higher in NA as a result of higher success rate at every step of the distribution process, illustrating the benefit of learning from a trial distribution before scaling up in other areas. Improvements in the first three steps relate to improvements in the system whereby registration was more complete (likely due to better training of registrars), community mobilization was strengthened, more distribution points were offered, and more nets were available for distribution to the target population. The high success in arrival at the distribution points when the child had been registered (CP) in both districts also indicates that caretakers had high interest in and willingness to receive a net. The final step, i.e. having the child sleeping under an LLIN, relies on the individual behaviour of the caretaker in adhering to the intervention. In this step, use was higher in NA than in MI even when comparing usage only among those who received an LLIN. This could be attributed to better information, education and communication (IEC) strategies in the distribution scale-up, shorter time from distribution (4 months vs. 9 months), and the season in which the distribution took place (rainy season vs. dry season). Since there is uncertainty as to which of these factors are responsible for the difference observed between the two districts, we do not attempt to conclude, but rather report on this difference.

The finding that the proportion of children sleeping under LLINs (58% in MI and 88% in NA) was higher than the accumulated system effectiveness in both districts can be explained by the fact that the success in the distribution steps are not mutually exclusive; hence even when failing in the first three steps of the distribution, a child could still end up sleeping under an LLIN by sharing a net with a sibling.

Children who received an LLIN were significantly more likely to use the net compared to children who did not receive an LLIN. This finding indicates that the community accepted the concept of using the nets for the specific child who it was intended for, in contrary to another study which showed that priority of sleeping under a bed net is given to older people [11]. In addition to the child receiving an LLIN, caretakers who perceived the LLINs to be better than conventional nets were also significantly more likely to use them for their children. The increase in usage due to the perception that LLINs are better than other nets emphasizes the importance of considering the community's preferences prior to a mass distribution and addressing the communities concerns through IEC campaigns. Although caretakers liking of the LLIN mesh size did not significantly influence the use of the nets, we found that, as previously seen in the Solomon Islands, the large mesh size of the Olyset^® ^net causes concern that mosquitoes may be able to penetrate the net [30].

Despite the high proportion of children who received an LLIN during the mass distribution scale-up among those who were already born at registration, the fact that some of these children were still missed during the distribution campaign strengthens the view that mass distributions should not be the exclusive mean of distribution. Additionally, there is an inherent problem with periodical targeted mass distributions, as newborns will always be missed. In this particular campaign, targeting pregnant women was a solution for some of these children, depending on the mother's attendance at the antenatal clinic, which is uncommon in early stages of the pregnancy. Thus, the "catch-up" and "keep-up" approaches, which allow for complimentary distributions to both rapidly increase ("catch-up") and also sustain ("keep-up") coverage, should be implemented [31-33]. "Keep-up" strategies may include continuous free distributions, targeted subsidies or voucher systems, depending on the community's willingness and ability to pay for LLINs.

The findings of this study demonstrate that free mass distributions can be a successful method for achieving equitable LLIN coverage. While the method using asset index has been questioned in relation to its relevance to household expenditure, it is commonly used as a tool to differentiate between socio-economic groups [9,17-19]. Inequities in health on national, regional, and local scales are a growing concern, and there is need to identify strategies to reduce the gaps between socio-economic groups [20]. In mainland Tanzania it has been shown that even within the rural setting, where households might be assumed to be of a uniformly poor socioeconomic status, inequities in health exist, influencing caretakers' care seeking behaviour and children's access to appropriate treatment [17]. Inequitable access to preventive measures for malaria have also been found, and despite Tanzania's social marketing efforts and voucher scheme, ITN coverage has been shown to be significantly lower in the poorest communities [15,18,19]. Nevertheless, a study in Kenya that assessed different delivery methods showed that a large-scale mass distribution of ITNs achieved equity [26].

## Conclusion

Targeted free mass distribution of LLINs can result in high and equitable bed net coverage among children under five. However, in order to sustain high effective use among both newborns and older children, there is need for complimentary distribution strategies between mass distribution campaigns. Considering the community's preferences prior to a mass distribution and addressing the communities concerns through information, education and communication, may improve the LLIN usage.

## Competing interests

The authors declare that they have no competing interests.

## Authors' contributions

NB - conception and design of the study, design of study tools, data collection, data management, analysis and interpretation of data, drafting the paper, revising the paper. ASA - conception and design of the study, design of study tools, analysis and interpretation of data, drafting the paper, revising the paper. DDS - analysis and interpretation of data, drafting the paper, revising the paper. AHA - design of study tools, data collection, data management, analysis and interpretation of data, drafting the paper. MR - conception and design of the study, design of study tools, revising the paper. AKA - design of study tools, data collection, drafting the paper. RSO - design of study tools, data collection, drafting the paper. AB - conception and design of the study, design of study tools, analysis and interpretation of data, drafting the paper, revising the paper. KK - conception and design of the study, design of study tools, analysis and interpretation of data, drafting the paper, revising the paper.
